# Experience of Early Postgraduate Transition to Intensive Care Medicine: A Phenomenological Study

**DOI:** 10.7759/cureus.64185

**Published:** 2024-07-09

**Authors:** Darragh Enright, Roslyn Colgan, Enda O'Connor

**Affiliations:** 1 Anaesthetics, University Hospital Galway, Galway, IRL; 2 Anaesthetics, Mater Misericordiae University Hospital, Dublin, IRL; 3 Anaesthesia and Critical Care, Saint James Hospital, Dublin, IRL

**Keywords:** postgraduate medical education (pgme), multiple and multidimensional transitions theory (mmt), phenomenology, clinical role transition, intensive care medicine

## Abstract

Background

Clinical role transition is known to pose a challenge to doctors in training. The intensive care unit (ICU) is noted to be a demanding workplace environment, although relatively little is known about the experience of doctors as they transition to intensive care medicine (ICM) at the postgraduate level. Thus, this study aimed to explore the experience of registrar-grade doctors undergoing this transition and to examine the interplay between personal and professional life at this time.

Methodology

This qualitative study was guided by transcendental phenomenology and multiple and multidimensional transitions theory (MMT). Data were collected via 11 semi-structured interviews and analysed using a six-step analysis. Peer debriefing and a reflexive diary were used.

Results

The experience of doctors undergoing the transition to ICM was found to be influenced by the clinical environment of the ICU, a demanding and potentially stressful workplace, and one in which collegial support was valued. The previous experience of the individual undergoing the transition was relevant, and the impact of this transition on their professional development and identity was notable. Consistent with MMT, the interplay between personal and professional life was significant, as participants outlined the impact of anxiety before a shift, the emotional and cognitive burden of a shift, and the effect of this transition on their personal relationships.

Conclusions

This study observes the ICU to be an extremely challenging workplace environment, with a notable influence on the personal lives of those working therein. Nevertheless, ICM offers invaluable opportunities for learning and both personal and professional growth.

## Introduction

Intensive care medicine (ICM) is a highly specialised and rapidly evolving field. An in-depth understanding of complex physiological and pathological processes is required, as well as the ability to manage acutely deteriorating patients and operate within a broad multidisciplinary group [1]. Clinical role transition can be a physically, mentally, and emotionally demanding time for doctors in training, who often feel ill-prepared for their new role [2,3]. Similarly, the transition from anaesthetic trainee, or physician trainee, to intensive care doctor may be a challenging one, posing the potential to threaten staff well-being and compromise patient care [4]. Contributory to this is a lack of confidence and preparedness for the management of advanced organ support among junior doctors [5], as well as the need to care for complex critically ill patients and the accompanying ethical dilemmas [6]. Notwithstanding the challenges around role transition when commencing intensive care unit (ICU) work and the influence they exert on professional and personal life, there are few studies on the experience of doctors as they transition into ICM at the postgraduate level. Thus, this qualitative study was designed.

Phenomenology is a research method that is used to explore and describe how people experience and make sense of the world around them, focusing on the lived experience of an individual. It holds the assumption that there is an essence (or essences) to shared experience [7] and aims to explore this from the perspective of those who have experienced it [8,9]. Phenomenology is an adaptable research approach that is well-suited to healthcare education [8] as it enables a more profound comprehension of the phenomenon under investigation. It is for this reason that it was chosen as the basis for this study examining the transition to ICM. Transcendental or descriptive phenomenology is a subset of the broader field of phenomenology. Utilised correctly, transcendental phenomenology is a rigorous research method that requires a systematic and disciplined approach [10]. It involves collecting rich, detailed descriptions of participants’ experiences and then analysing these descriptions to identify common themes and patterns. In transcendental phenomenology, no prior assumptions should inform phenomenological inquiry [8]; accordingly, researcher bias and prior beliefs are suspended in transcendental phenomenology. Furthermore, as this study is an example of insider research, transcendental phenomenology was preferred to hermeneutic or interpretive phenomenology to allow for a more objective analysis of the participants’ experiences [8].

Multiple and multidimensional transitions theory (MMT) was selected as the theoretical framework for this study. Jindal-Snape [11] proposed MMT in 2016, and it has previously been utilised to examine clinical role transition [2,12]. MMT observes transition to be a dynamic, non-linear process, incorporating multiple domains (physical, psychological, social, cultural) and contexts (role, workplace, home, education) [2,11,12]. Each of these domains is interconnected, exerting influence on each other and the experience of the transition as a whole [2]. Moreover, the transition may impact the lives of significant others, and vice versa [12]. Clinical role transition has been correlated with increased rates of burnout [13,14], with burnout noted to be an issue facing ICM [15,16]. Thus, attaining a detailed understanding of the transition to ICM is likely a worthwhile endeavour. MMT may help foster an understanding of the various challenges and opportunities that people face during the transition, as well as the diverse ways in which individuals experience and cope with transition. In this way, MMT can inform intervention as it helps individuals, organizations, and policymakers better understand the complexities of transition and develop more effective strategies to support individuals and communities during periods of change. As MMT acknowledges the intricate and multifaceted nature of transition [12], it was chosen as the conceptual basis for this study to allow for a more holistic understanding of the transition to clinical ICM and to explore the interplay between an individual’s various domains and contexts during this time.

Utilising transcendental phenomenology and MMT, the purpose of this study was to answer the following research questions: how do registrar-grade doctors describe their transition to ICM? To what extent does the transition to ICM impact the personal lives of those doctors undergoing this transition?

## Materials and methods

This study was conducted at the University of Galway. Ethical approval was obtained from the Galway Clinical Research Ethics Committee, in accordance with the World Medical Association Declaration of Helsinki. Informed consent was obtained from all participants for both participation in the study and the publication of its findings. Critical case sampling was utilised to select participants. The sampling frame consisted of anaesthetic and physician trainees who had undertaken clinical rotations of at least two months in Irish ICUs. To allow for greater transferability of findings, only those who had made the transition to ICM before the COVID-19 pandemic were included. Invitation to participate was via email from a third party not directly involved in the study, distributed via individual intensive care departments. Candidates were recruited from multiple centres. Sample size was guided by principles of data saturation, facilitated by contemporaneous data analysis.

Data collection was via individual interviews, allowing the researcher to gain in-depth information and personal opinions from participants [17]. Interviews were conducted virtually via online video-call software. Semi-structured interviews were utilised to allow for a focused, time-efficient discussion [18] and to balance the need for a theory-driven deductive enquiry with the capacity to discover unanticipated, emergent data [19]. A potential weakness of this approach is that interviewees may not have the opportunity to express their own unique perspectives [18]. To counteract this, guiding questions were open-ended, and the interviewer acted as a neutral observer rather than expressing any approval or disapproval.

Initial questions were open-ended and intended to stimulate unstructured inquiry. Further questions were intended to stimulate more in-depth reflection into the transition process being studied, before closing questions sought to gain an overview or overall impression of the experience of the participants. These questions used to guide the interview process are outlined in Table 1. These were marginally amended after the analysis of two pilot interviews, which were included in the data analysis process. With the consent of the participants, interviews were recorded to facilitate transcription.

**Table 1:** Questions used to guide the semi-structured interviews.

Questions used to guide semi-structured interview
Can you tell me about your experience of your transition to intensive care medicine (ICM)?
Do you recall your first night or first few nights on call? How did you feel during them?
What did you find most challenging during your first month in ICM?
What did you find most rewarding during your first month in ICM?
Was the transition as you expected or were there any aspects of it that surprised you?
Do you feel the transition to ICM affected other aspects of your life?
Do you feel like other aspects of your life impacted your transition to ICM?
Do you think this transition to ICM affected your self-identity, either as a doctor and/or as a person?
Do you reflect on your transition to ICM as a positive or negative experience, overall?
Are there any other points you would like to discuss?

Analysis was guided by the framework of Giorgi [20], whereby a researcher first studies individual examples of a larger phenomenon before progressing to a more universal understanding of that phenomenon. Data analysis software (nVivo) was used to assist the process. Constant comparative analysis was used, whereby the information gathered was coded, after each interview, into emergent themes or codes. These data were then revisited after initial coding until it was evident that no new themes were emerging [21]. This approach allowed for sample guidance, as well as the identification of emerging themes and the point of data saturation. Interview transcripts were studied in detail and a summary form of each transcript was created as a means of data condensation [22].

Several methods were implemented to ensure rigour in this study. Member checking, an audit trail [23], and peer debriefing were utilised. The transferability of the research findings was aided by the recruitment of participants from different specialities and from several different clinical sites. As the primary researcher is an anaesthetic trainee, this study is an example of ‘insider research’. ‘Epoché,’ or ‘bracketing’ was practised, and a reflexive diary was maintained by the primary researcher to reduce the risk of bias impacting the study [24]. An experienced external research supervisor was also involved in the study to ensure research credibility.

## Results

**Figure 1:**
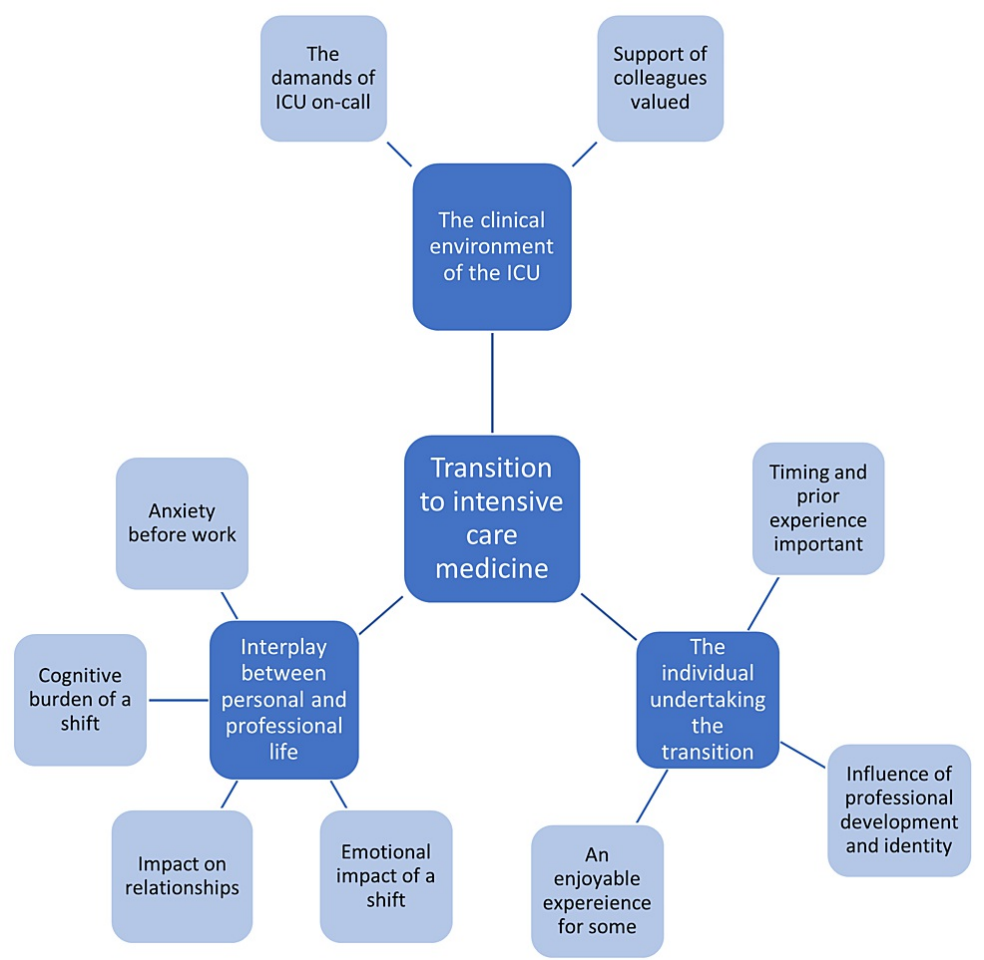
The key themes (dark blue boxes, white font) and subthemes (light blue boxes, black font) identified.

In total, 11 participants were interviewed (nine anaesthetic trainees and two physician trainees; five females and six males) between March and May 2022 (duration, 18-36 minutes; average duration, 28 minutes). Each had undergone the transition to ICM in several different ICUs in different geographical regions at various stages of their career. This work’s results are presented thematically in narrative format. Participants have been denominated P1-P11, with their clinical role at the time of the interview defined in Table 2. Three key themes were identified, which, along with their subthemes, are outlined in Figure 1.

**Table 2:** Clinical role of the participants at the time of interview.

Participant	Clinical role at the time of the interview
P1	Specialist Anaesthetic Trainee, Year 5 (SAT 5)
P2	Medical (Respiratory) Registrar, Year 4
P3	SAT 6
P4	SAT 6
P5	SAT 5
P6	Medical (Infectious Diseases) Registrar, Year 4
P7	SAT 5
P8	Paediatric Anaesthesia Fellow
P9	SAT 5
P10	SAT 5
P11	SAT 6

Key theme 1: the clinical environment of the ICU

The potentially stressful nature of the transition to ICM was most evident when participants spoke about the demands of their first call shifts. ‘I definitely remember having a tremor, headache, migraine - all the functional symptoms of stress and anxiety, waiting for that bleep to go off, and every bleep you’d be jumping out of your skin’ - P8. ‘ [I felt] horrible … absolutely out of my depth…You’re living in complete fear that something will come through the door… and that you won’t be able to handle it’ - P5. The significant workload posed a challenge for many participants. This workload extended beyond the ICU to hospital wards and emergency departments. ‘ICU call really is relentless … You’re supposed to look after your own patients - the ICU patients - but everyone else in the hospital wants you to look after theirs as well’ - P3. In some instances, the taxing nature of the transition to ICM resulted in participants displaying features of depersonalisation and burnout, forcing them to reflect upon their career choices. ‘The patients are becoming not people to you anymore, but just something you do as part of your job. It makes you think… “Do I need to hold back or change or, you know? Do something different”’ - P9.

The support of colleagues during the transition was highly valued. This included support from senior consultant colleagues and from nursing and allied health colleagues. ‘Any time I reached out to ask for help or assistance, I got it … It was always supportive’ - P1. ‘I felt there was a lot of support … there was a little bit more shared decision making … There was a multi-disciplinary team there and they were able to direct you’ - P9. The subthemes and supporting quotes for key theme 1 are displayed in Table 3.

**Table 3:** The subthemes and supportive quotes of key theme 1: clinical environment of the ICU.

Subtheme	Supporting quotation(s)
The demands of ICU on-call	‘I definitely remember having a tremor, headache, migraine - all the functional symptoms of stress and anxiety, waiting for that bleep to go off, and every bleep you’d be jumping out of your skin’ – P8.
	‘[I felt] horrible … absolutely out of my depth … You’re living in complete fear that something will come through the door … and that you won’t be able to handle it’ – P5
	‘ICU call really is relentless … You’re supposed to look after your own patients - the ICU patients - but everyone else in the hospital wants you to look after theirs as well’ – P3
	‘The patients are becoming not people to you anymore, but just something you do as part of your job. It makes you think … “Do I need to hold back or change or, you know? Do something different”’ – P9
Support of colleagues	‘Any time I reached out to ask for help or assistance, I got it … It was always supportive’ – P1
	‘I felt there was a lot of support … there was a little bit more shared decision making … There was a multi-disciplinary team there and they were able to direct you’ – P9

Key theme 2: the individual undertaking the transition

A factor that influenced trainees’ experience of ICM transition was its timing relative to their stage of training, and thus their prior experience, or lack thereof, of intensive care practice. Appropriate timing of the transition was felt to potentially ‘minimise that level of feeling completely out of your depth’ - P11. ‘It came at the right time of my training … I didn’t feel as if I was unprepared going into it’ - P9. A lack of preparedness was noted by some Anaesthetic Trainees, who felt that they were ‘rushed’ (P5) to transition to ICM before they were ready to do so. ‘I didn’t really feel like I was trained to work in ICU. I didn’t feel like the exams prepared me in any way for ICU’ - P1. ‘I just feel like it was very early for me to be taking on that responsibility, and I was nervous, and stressed’ - P11. Conversely, physician trainees tended to transition to ICM at a later stage in their training, as more experienced clinicians, a factor noted to facilitate the transition process.

The transition to ICM was influenced by one’s professional development and identity, and vice versa. This transition was initially observed to entail an acute upskilling process. A large volume of learning occurred in a relatively short period of time, described as a ‘baptism of fire’ (P7). While challenging, this also presented an opportunity for professional development, and participants reported a sense of achievement in their new role. ‘There’s a self-aggrandisement. You nearly say, “Oh cool, I did it. The sickest people in the hospital - I got them all through’” - P5. The transition to ICM also promoted reflection about perceived professional identity. ‘There’s a bit of proving yourself. Whether it’s proving yourself to yourself or proving yourself to your colleagues and your consultants, but you want to be seen to be good, to be taking that big step up’ - P1.

Despite the challenges outlined above, six of the eleven participants described the transition to ICM as a positive experience overall. It was described as ‘collaborative’ (P2), ‘social’ (P5), and ‘exciting’ (P5). Others described their enjoyment of this transition. ‘I really enjoyed working in the ICU … I started into it and thought “this is something I must do. I hope I’m good enough”, and then it became something that I enjoy doing’ - P10. The subthemes and supporting quotes for key theme 2 are displayed in Table 4.

**Table 4:** The subthemes and supportive quotes of key theme 2: the individual undertaking the transition.

Subtheme	Supporting quotation(s)
Timing of the transition and prior experience	‘It came at the right time of my training … I didn’t feel as if I was unprepared going into it’ – P9
	‘I didn’t really feel like I was trained to work in ICU. I didn’t feel like the exams prepared me in any way for ICU’ – P1
	‘I just feel like it was very early for me to be taking on that responsibility, and I was nervous, and stressed” – P11
Professional development and identity	‘There’s a self-aggrandisement. You nearly say, “Oh cool, I did it. The sickest people in the hospital - I got them all through”’ – P5
	‘There’s a bit of proving yourself. Whether it’s proving yourself to yourself or proving yourself to your colleagues and your consultants, but you want to be seen to be good, to be taking that big step up’ – P1
An enjoyable experience	‘I really enjoyed working in the ICU … I started into it and thought “this is something I must do. I hope I’m good enough”, and then it became something that I enjoy doing” – P10

Key theme 3: interplay between work and personal life

One way in which the transition to ICM impacted the personal life of doctors was by inducing anxiety prior to a shift. ‘You’re anxious and you’re not in great form a lot of the days pre-call, thinking about the call shift ahead’ - P1. The nature of working in ICM entailed a cognitive burden that affected participants’ personal as well as professional lives. ‘Your job definitely affects your personal life … you’re dealing with ethical issues that you can’t really discuss with other people’ - P3.

The emotional impact of a challenging shift also impacted participants’ personal lives. ‘You might be going over decisions, or bad outcomes, dealing with the emotional hangover from the shift’ - P1. ‘It was tough at the time, and it was tough going home from those difficult shifts and replaying situations in my head, and second guessing myself’ - P1. In some instances, one particularly traumatic case or poor outcome had a lasting emotional impact on participants. The high acuity of ICM contributed to the disruption of participants’ sleep cycle, which again impacted them beyond that shift. ‘One on-call that you could have … could affect you for two weeks afterwards in terms of your sleep cycle because of the adrenaline rush of managing really severe, critically ill patients’ - P9. This interplay between professional and personal life was another factor that resulted in participants displaying signs of emotional exhaustion, causing them to reflect on their career choices. ‘I definitely think it really affected me outside of work … there were definitely times where I thought “I can’t do this anymore” … I felt completely depleted’ - P11.

The transition to ICM was seen to affect the interpersonal relationships of the participants. ‘Absolutely it does have a knock-on effect on your personal life as well, and your interpersonal relations - your potential to go home being well rested to see your family’ - P9. ‘You’d spend the day before [a call shift] stressing about it … This stress filters through to your partner and your friends’ - P1. To a lesser extent, the lives of participants outside of work were reported to have affected their professional lives. Moving to an unfamiliar city for work with the inherent loss of a social support network was one reason cited for this. Another reason that was highlighted pertained to the demands of caring for young children which hindered the ability to rest and further impacted an already-disrupted sleep cycle. The subthemes and supporting quotes for key theme 3 are displayed in Table 5.

**Table 5:** The subthemes and supportive quotes of key theme 3: interplay between work and personal life.

Subtheme	Supporting quotation(s)
Anxiety prior to a shift	‘You’re anxious and you’re not in great form a lot of the days pre-call, thinking about the call shift ahead’ – P1
Cognitive burden of a shift	‘Your job definitely affects your personal life … you’re dealing with ethical issues that you can’t really discuss with other people’ – P3
Emotional impact of a shift	‘You might be going over decisions, or bad outcomes, dealing with the emotional hangover from the shift’ – P1. ‘It was tough at the time, and it was tough going home from those difficult shifts and replaying situations in my head, and second guessing myself’ – P1
Impact on relationships	‘Absolutely it does have a knock-on effect on your personal life as well, and your interpersonal relations - your potential to go home being well rested to see your family’ – P9. ‘You’d spend the day before [a call shift] stressing about it … This stress filters through to your partner and your friends’ – P1

## Discussion

This study has found that the experience of trainees transitioning to ICM practice is influenced by the ICU environment, the individual undergoing the transition, and the significant interplay between the personal and professional lives of the participants. It is interesting to observe, however, that while most participants describe the transition to ICM to be an extremely challenging one, it is ultimately reflected on as a worthwhile endeavour by most. The demanding nature of the transition and its profound impact on the personal lives of participants is seemingly balanced by the satisfaction attained from the new role, when viewed retrospectively, and the inherent personal growth and professional development.

The significant interplay between the professional and personal lives of participants is consistent with MMT. Participants spoke about the physical manifestations of anxiety as well as the effects of sleep deprivation during the transition to ICM (physical domain). They also discussed the cognitive and emotional burden of ICM (psychological domain). Gordan et al. [12], when utilising MMT to examine the transition from student to doctor, had previously highlighted the importance of interpersonal relationships in facilitating successful transition as well as the ability of transition to influence relationships. Participants in this study outlined the impact of the transition to ICM on their ability to maintain healthy personal relationships, which is a feature of the social domain of MMT. Thus, MMT would seem to be an appropriate lens through which to view this transition to ICM.

While it may not be entirely surprising to learn that the transition to such a high acuity specialty poses a challenge, the extent of this challenge reported by trainees is noteworthy. The findings of this study suggest that appropriate timing and preparation are crucial for trainees’ experiences of the transition to ICM. Thus, attempts to mitigate the challenging nature of this transition may target the level of ill-preparedness reported by trainees and its timing relative to their level of training. Indeed, previous research has shown preparation before clinical role transition to be influential in the transition process [15]. The signs of burnout displayed by some respondents were a concerning finding of this study. Kok [19] had previously highlighted the impact of burnout on ICU professionals and patient care. The results of this study highlight the importance of supporting healthcare professionals during the transition to ICM and throughout their careers. Strategies such as mentorship, debriefing, and support for self-care can help mitigate the stress and pressure of the job and prevent burnout [25]. This study also suggests the need for greater recognition of the challenges faced by healthcare professionals in this field and the importance of addressing them to ensure the maintenance of staff morale and delivery of high-quality patient care.

The findings of this study are largely consistent with previous studies that broach the subject of clinical role transition and early exposure to clinical ICM. Brown et al. [2] and Sturman et al. [3] had previously described the challenging nature of clinical role transition. The results of this study are also consistent with those of Yardley et al. [26], who noted how the stressful nature of role transition prompted a re-examination of one’s professional identity. Other research has demonstrated how ICM is a demanding field in which to work. Heffner et al. [4] discussed the heavy workloads and pressurised training environment of ICM and commented on the detrimental effect of fatigue on staff morale and performance. These findings are also comparable with the results of this study. The ability of working in ICM to impact the physical and mental well-being of junior doctors, with the resultant risk of burnout and moral injury, was outlined by Coughlan et al. [1]. This study, similarly, outlines the cognitive and emotional burden of a shift in ICU, which may impact the psychological well-being of trainees, while the sleep deprivation associated with such a high-acuity and demanding specialty may negatively impact physical health. Pnevmatikos [6] and Curtis [27] discuss the rewarding aspects of ICM, which involves using diverse skills to care for complex critically unwell patients, often with satisfying outcomes. This echoes the sentiments of the participants of this study, who ultimately felt that ICM was a rewarding field in which to work.

Social cognitive theory (SCT) would have been an alternative theoretical basis for this study. Bandura [28] describes how SCT views learning as the consequence of an individual’s interpretation of and interaction with an environment. Participants’ description in this study of the value of helpful colleagues and a supportive learning environment is in keeping with SCT. The varying degree of stress reported by participants is also consistent with SCT, in that the characteristics and agency of the individual (their ability to manage stress) are seen to impact the learning experience. The timing of learning is not usually a feature of SCT, but its importance was a notable finding of this study. Indeed, as SCT notes that the traits of the individual learner influence the learning process, and these characteristics are likely to change with time, it is reasonable to expect that the timing of this learning process will be influential.

There are limitations to this study. The sample size of 11 participants was relatively small. Despite this, the study ‘offers new insights that contribute substantially to or challenge current understandings’, a criterion used by Malterud et al. [29] to describe an adequately powered qualitative study. Additionally, contemporaneous data analysis allowed for the identification of the point of data saturation. Most participants had undergone the transition to ICM several years before the interview, which could be argued to affect the accuracy of their reflections. However, this ensured that the transition to ICM was made before the COVID-19 pandemic and enhanced the transferability of the findings, rather than being reflective only of working in ICM during this unique event. The timing between the transition and interview also allowed participants to reflect more wholly on the transition and its wider impact on them as doctors and as individuals. As this study is an example of insider research, there was a risk of bias influencing participant recruitment, data collection, and data analysis and interpretation [30]. Third-party recruitment was utilised to help minimise the risk of bias influencing participant recruitment. A reflexive diary was used to assist the process of bracketing and minimise the risk of bias on the part of the primary researcher impacting data collection and interpretation. Peer debriefing was undertaken by an experienced clinical researcher, though one who is also a consultant intensivist and could also be considered an insider in the context of this study. This process resulted in a slight amendment of the questions used for data collection following the initial review of transcripts and helped limit bias impacting data analysis.

## Conclusions

In summary, this study reinforces ICM as an extremely challenging workplace environment, but one that offers a valuable opportunity for learning and both personal and professional growth. It is recommended that doctors undergoing this difficult transition do so at a time that feels appropriate to them, that they receive support from senior colleagues, and that training is targeted to those commencing ICM practice, particularly in an on-call setting.
